# Feasibility of a Novel Quantum Communication Protocol in Jerlov Type I Water

**DOI:** 10.3390/e25010016

**Published:** 2022-12-22

**Authors:** Alessia Allevi, Maria Bondani

**Affiliations:** 1 Department of Science and High Technology, University of Insubria and Institute for Photonics and Nanotechnologies, IFN-CNR, Via Valleggio 11, I-22100 Como, Italy; 2 Institute for Photonics and Nanotechnologies, IFN-CNR, Via Valleggio 11, I-22100 Como, Italy

**Keywords:** quantum states of light, quantum communication, photon-number-resolving detectors

## Abstract

Underwater communication based on the use of optical quantum resources has attracted a lot of attention in the last five years due to the potential advantages offered by quantum states of light. In this context, we propose to operate in the mesoscopic intensity regime, where the optical states are well populated and the employed detectors have photon-number resolution. By exploiting these features, we demonstrate that a novel communication protocol based on the experimental quantification of nonclassicality of mesoscopic twin-beam states can be used to transmit binary signals encoded in two single-mode pseudothermal states with different mean values through a Jerlov type I water channel. The experimental results are in perfect agreement with the developed theoretical model, and the feasibility of the protocol is also investigated as a function of the data samples corresponding to each one of the two signals. The good quality of the results encourages a more realistic implementation of the protocol, also exploring the maximum distance at which the quantum states remain nonclassical and thus can be still properly discriminated.

## 1. Introduction

Underwater communications are traditionally implemented by exploiting acoustic sources, which allow the propagation through kilometers of water [1,2]. However, the rate at which the communication protocols are performed is quite low due to the velocity of sound in water. This makes the transmission of information not completely secure. At variance with this scenario, some years ago, new experimental strategies exploiting optical states, both classical and quantum, have been proposed and tested [3]. While the standard strategy is based on weak coherent states of light, in the last five years, many experiments have involved quantum resources, such as entangled states, because they can offer a higher level of security, even though they are more fragile than coherent states [4,5,6,7,8,9,10]. In these cases, the rate at which information is transmitted is definitely higher thanks to the use of electromagnetic waves instead of acoustic ones. However, the maximum distance at which the protocols work is definitely shorter because of the different kinds of optical losses, such as absorption, diffusion, and depolarization affecting the system [11]. Moreover, the amount of losses strongly depends on the wavelength of the employed light source and on the type of water. Indeed, oceanic and coastal water can be classified into different classes according to Jerlov classification [12]. In particular, Jerlov’s oceanic water types (I to III) correspond to waters mostly free of inorganic particles and extra-colored dissolved organic material from terrigenous sources [13,14], while Jerlov’s coastal water types (IC through 9C) result from both biological processes and also a variety of nonbiological processes. Despite the losses due to water, the experimental results achieved in the optical domain are really promising for communication protocols in which the communication links are up to hundreds of meters long.

Most experimental implementations were made so far at the single-photon level by encoding information in the different degrees of freedom of photons, such as polarization [4,14] and orbital angular momentum [9]. At variance with these realizations, we have recently proposed a different scenario, in which mesoscopic quantum states of light were detected by photon-number-resolving detectors [15,16]. In particular, we considered multi-mode twin-beam (TWB) states entangled in the number of photons [17,18,19]. We have demonstrated that in the case in which one arm of the TWB is transmitted through water and the other one propagates in free space, we can still observe nonclassical correlations at moderate distances. At the same time, we have proposed a novel communication protocol, based on TWB states and on the calculation of nonclassical correlations in terms of the noise reduction factor [17], in which the information is encoded in a binary pseudothermal signal superimposed on the TWB and the losses mimic an eavesdropper’s interference [20]. By taking advantage of all these investigations, in this work, we present a study based both on theoretical and experimental results aimed at demonstrating that our communication protocol can be succesfully implemented in the underwater environment. In particular, we investigate the feasibility of the protocol as a function of the number of repetitions required to distinguish the encoded information. The good quality of the results encourages a more realistic implementation of the protocol, also exploring the maximum distance at which the quantum states remain nonclassical.

## 2. Materials and Methods

Multi-mode TWB states entangled in the number of photons are described by the following density matrix [21],

(1) $$\rho_{\mathrm{TWB}}^{\mu }=\sum_{n=0}^{\infty }P^{\mu }\left(n\right)|n,n\rangle \langle n,n|,$$

in which $|n\rangle =\delta \left(n-\sum_{k=1}^{\mu }n_{k}\right){⨂}_{k=1}^{\mu }{|n_{k}\rangle }_{k}$ and *n* is the overall number of photons in the μ spatio-spectral modes that impinge on the detector, while $P^{\mu }\left(n\right)$ is the multi-mode thermal distribution [22]

(2) Pμ(n)=n+μ−1n〈n〉μn1+〈n〉μ−n−μ

where 〈n〉 is the mean number of photons in each arm. A sufficient criterion for entanglement, which is also experimentally accessible, is associated to the calculation of the noise reduction factor, which is defined as [17]

(3) $$R=\frac{\sigma^{2}\left(n_{1}-n_{2}\right)}{\langle n_{1}\rangle +\langle n_{2}\rangle },$$

where $\sigma^{2}\left(n_{1}-n_{2}\right)$ is the variance of the distribution of the photon-number difference between the two parties and $\langle n_{1}\rangle +\langle n_{2}\rangle$ is the shot-noise level. Values of *R* less than unity prove the existence of sub-shot-noise, i.e., nonclassical, correlations. Recently, we have shown that in the case of multi-mode TWB states, the noise reduction factor can be also written in terms of measurable quantities, such as detected photons, and it can also include the effect of losses and noise sources [23]. In particular, *R* can be written as

(4) $$R=1-\frac{2\eta t\langle m\rangle }{\left(1+t\right)\langle m\rangle +\langle m_{N}\rangle }+\frac{{\left(1-t\right)}^{2}{\langle m\rangle }^{2}}{\mu \left[\left(1+t\right)\langle m\rangle +\langle m_{N}\rangle \right]}+\frac{\sigma^{2}\left(m_{N}\right)-\langle m_{N}\rangle }{\left(1+t\right)\langle m\rangle +\langle m_{N}\rangle },$$

where 〈m〉 is the mean number of detected photons in a TWB arm, η is the global quantum efficiency of the detection system, and *t* is the transmission efficiency quantifying the balancing between the two arms, while $\langle m_{N}\rangle$ and $\sigma^{2}\left(m_{N}\right)$ are the mean value and the variance of the noise source, respectively. In particular, in the case of a single-mode pseudothermal noise $\sigma^{2}\left(m_{N}\right)=\langle m_{N}\rangle \left(\langle m_{N}\rangle +1\right)$, so that Equation (4) reduces to

(5) $$R=1-\frac{2\eta t\langle m\rangle }{\left(1+t\right)\langle m\rangle +\langle m_{N}\rangle }+\frac{{\left(1-t\right)}^{2}{\langle m\rangle }^{2}}{\mu \left[\left(1+t\right)\langle m\rangle +\langle m_{N}\rangle \right]}+\frac{{\langle m_{N}\rangle }^{2}}{\left(1+t\right)\langle m\rangle +\langle m_{N}\rangle }.$$

If one arm of the TWB propagates in water, and the other arm is transmitted in free space, the two arms are imbalanced mostly due to water absorption but also due to the possible presence of optical elements inserted in only one of the two arms [15]. The amount of losses limits the maximum distance at which the TWB states can propagate while maintaining their nonclassical character [24,25]. Thus, in view of implementing an experimental realization of underwater communication, it is of crucial importance to investigate the effect of losses and noise on the nonclassicality of the employed quantum states. First of all, we consider the case in which there are no noise sources, and one arm of the TWB is transmitted through different Jerlov types of water. In particular, we assume that the losses are only due to water absorption and are modeled as $t=10^{-\alpha x}$, where α is the absorption coefficient per unit length [12]. Since the experimental implementation presented in the next section has involved light pulses at 523 nm, the following theoretical studies consider the same wavelength.

**Figure 1:**
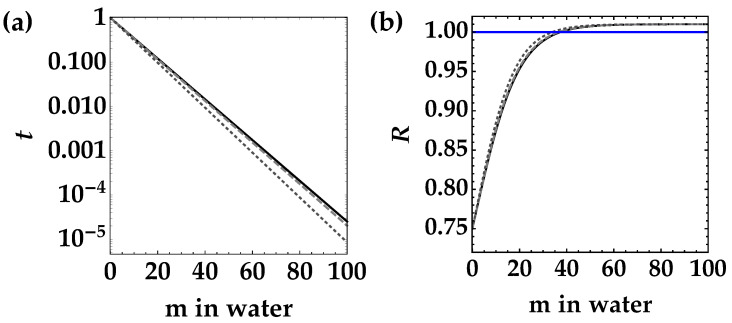
Transmission coefficient *t* (panel (**a**)) and noise reduction factor *R* (panel (**b**)) as a function of the distance traveled in water for Jerlov water types from I to III. In panel (**b**), we set 〈m〉=1, μ=100, and η=0.25. Black solid curves correspond to type I water (α = 0.0460 m${}^{-1}$), gray dashed curves correspond to type II (α= 0.0469 m${}^{-1}$), and dark-gray dotted curves correspond to type III (α= 0.0507 m${}^{-1}$). The transmission coefficient is shown in semi-log scale. The blue straight line in panel (**b**) corresponds to the condition R=1.

In Figure 1, we plot the value of *t* (panel (a)) and the corresponding noise reduction factor (panel (b)) as a function of the path in water for Jerlov water types from I to III. First of all, we notice that the curves are almost undistinguishable, since the values of α are really similar to each other, as explicitly indicated [12] in the caption of Figure 1. Secondly, we emphasize that there is a maximum propagation distance within which the noise reduction factor remains below 1. In principle, this value can be increased by changing the wavelength of light. For instance, by shifting the light to the blue region, where the typical values of α are ten times smaller than in the green region, hundreds of meters of water propagation are possible [13].

As a second kind of theoretical investigation, we study the behavior of *R* for a given choice of Jerlov water type, e.g., type I, as a function of the mean number of photons, 〈m〉, of the TWB and of the propagation path in water of one of the arms.

**Figure 2:**
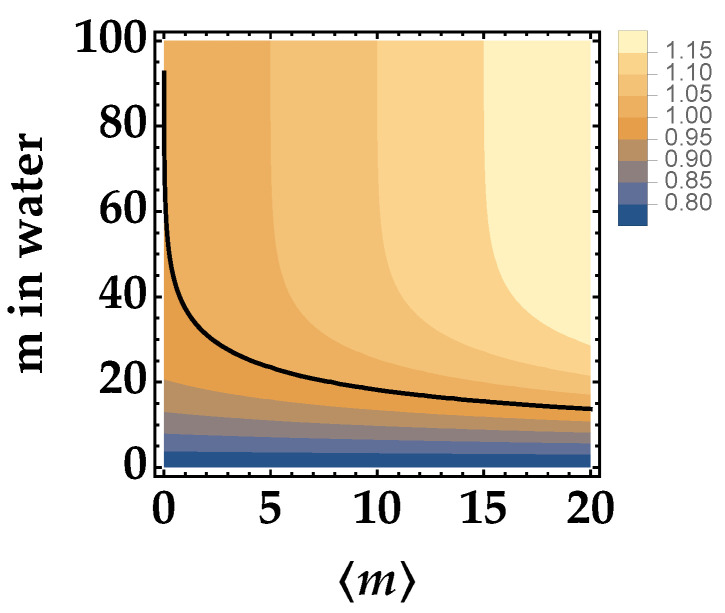
Noise reduction factor *R* as a function of the mean number of photons in the TWB arm and of the distance traveled in Jerlov type I water of just one arm for μ=100, η=0.25, and $\langle m_{N}\rangle =0$. The black curve corresponds to the condition R=1.

The results are shown in Figure 2 as a contour plot according to Equation (5), in which *t* is still modeled as $10^{-\alpha x}$, and for $\langle m_{N}\rangle =0$, that is for a zero-mean noise source. Indeed, when $\langle m_{N}\rangle$ is different from 0, the situation changes. In particular, in view of implementing the communication protocol mentioned in the Introduction [20], it is interesting to investigate what happens when a small contribution of pseudothermal noise is added to one TWB arm.

**Figure 3:**
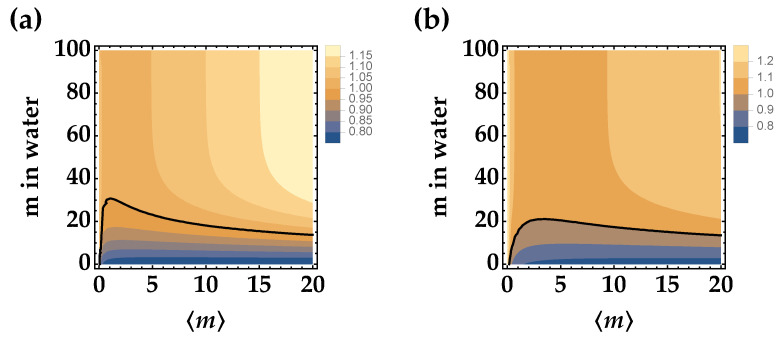
Noise reduction factor *R* as a function of the mean number of photons in the TWB arm and of the distance traveled in Jerlov type I water of just one arm for $\langle m_{N}\rangle =0.1$ (panel (**a**)) and $\langle m_{N}\rangle =0.3$ (panel (**b**)). In both panels, we set μ=100 and η=0.25, and the black curve corresponds to the condition R=1.

In Figure 3, we show the contour plots for $\langle m_{N}\rangle =0.1$ (panel (a)) and $\langle m_{N}\rangle =0.3$ (panel (b)). We can notice that adding a noise source makes the observation of nonclassical correlations more difficult, even if the larger the mean value of TWB, the smaller the value of *R*.

Thanks to the results obtained with these theoretical investigations, we can conclude that there are conditions under which it is still possible to observe nonclassical correlations even in the presence of a thermal noise and also for moderate propagation distances in water. We thus continue our investigation also from the experimental point of view.

**Figure 4:**
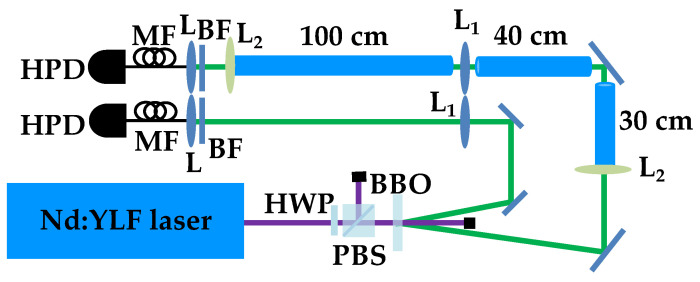
Sketch of the experimental setup. HWP: half-wave plate; PBS: polarizing cube beam splitter; BBO: β-barium borate crystal; L_1_: 1000 mm focal length lens; L_2_: 2000 mm focal length lens; BF: band-pass filter; L: achromatic doublet; MF: multi-mode optical fiber; HPD: hybrid photodetector. See the text for details.

To this aim, in Figure 4, we show the implemented experimental setup.

The fourth harmonics (at 262 nm) of a Nd:YLF laser regeneratively amplified at 500 Hz is sent to a β-barium borate crystal to generate the process of parametric downconversion and produce mesoscopic TWB states at frequency degeneracy, that is at 523 nm. One arm of the TWB is transmitted in free space for a global distance equal to 201 cm, while the other arm propagates partly in free space and partly in water. In particular, the propagation in water is 170 cm long and is made possible by the use of three plastic tubes 30 cm, 40 cm and 100 cm long filled with tap water (Jerlov type I water). Water is maintained in each tube by means of either laser windows or lenses with long focal length (2 m). Note that the use of optical elements must be kept into account in the calculation of the transmission coefficient *t* appearing in Equation (5). Since both arms of TWB are moderately long, in our setup, two 1 m focal-length lenses are used in order to keep under control the natural divergence of TWB [16]. These two lenses are placed in the two arms at 1 m from each collection system, which is composed by an achromatic doublet and a multi-mode fiber (with 600 μm core diameter) delivering the light to the photon-number-resolving (PNR) detector. In particular, we use a pair of commercial hybrid photodetectors (HPDs, mod. R10467U-40, Hamamatsu Photonics) operated at room temperature. Each detector output is then amplified, synchronously integrated, and digitized (ADC, PCI-6251, National Instruments). To convert the output voltages of the detection chain into numbers of detected photons [26,27,28], we apply the self-consistent method described in [29]. The method consists of modeling the detection process, which for the class of detectors we are using is composed of two steps: photodetection by the photocathode and amplification. The first process is described by a Bernoullian convolution, whereas the second one can be well approximated by the multiplication by a constant gain factor, γ [30]. The value of γ can be obtained by measuring the light at different values of the overall detection efficiency η of the apparatus. Once the value of the gain is determined, we have direct access to the shot-by-shot number of detected photons and can thus evaluate the statistical properties of the measured states [31]. In the experiment, the energy of the pump beam is changed by rotating a half-wave plate followed by a polarizing cube beam splitter. A sequence of 100,000 laser shots is saved for each choice of rotation angle.

## 3. Results

**Figure 5:**
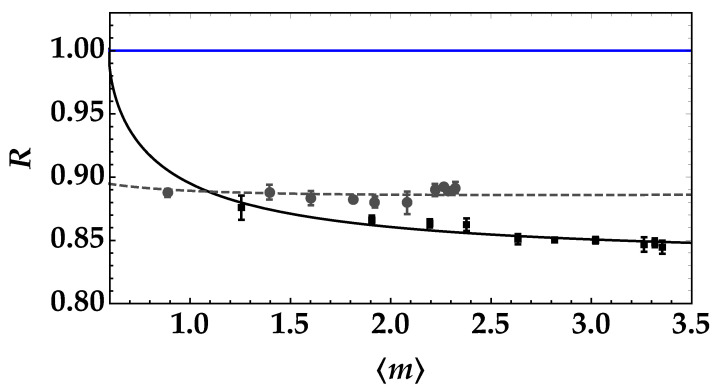
Noise reduction factor as a function of the mean number of detected photons in the TWB arm in the absence of tubes (black squares + error bars) and in the presence of tubes filled with water (gray dots + error bars). The theoretical fitting function according to Equation (4) is superimposed on each data set with the same color choice. The values of the fitting parameters for black data are: η=0.167±0.004 and $\langle m_{N}\rangle =0.59\pm 0.08$, while those corresponding to gray data are: t=0.56±0.02 and $\langle m_{N}\rangle =0.13\pm 0.06$. The blue straight line corresponds to the condition R=1.

As anticipated in the previous section, the nonclassicality of the TWB states can be investigated taking into accunt the difference between the propagation channels, that is considering the losses in the arm that propagates in water due to both water absorption and optical elements placed at the endings of the plastic tubes. The measured noise reduction factor is shown in Figure 5 as a function of the mean number of photons in one TWB arm in the absence of tubes (black squares + error bars) and in the case of tubes filled with water (gray dots + error bars). The data are shown together with the corresponding theoretical fitting functions according to Equation (4). In general, we can observe that all the data are well below the nonclassicality threshold R=1, thus proving that the measured states are nonclassically correlated. Note that in both cases, a Poissonian noise source due to dark counts or some spurious light was included in the fitting functions (in Equation (4), we assume $\sigma^{2}\left(m_{N}\right)=\langle m_{N}\rangle$). We note that this Poissonian noise contribution is larger in the absence of tubes, while it is much lower in the presence of tubes filled with water. Indeed, the presence of tubes reduces the possibility of detecting spurious light, since they shield it, thus avoiding its collection.

**Figure 6:**
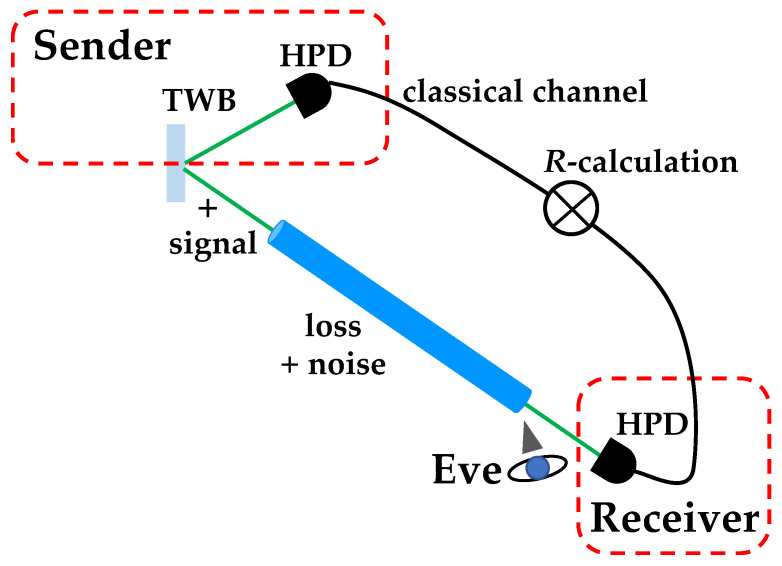
Sketch of the optical communication link, in which a binary thermal noise signal is superimposed on the portion of TWB propagating in water, while the other portion of TWB propagates in free space. By evaluating the noise reduction factor, it is possible to discriminate which thermal noise signal has been sent and, at the same time, to determine the presence or not of an eavesdropper’s attack.

In the absence of tubes, we can exctract the value of η from the fit by assuming t=1, while in the presence of tubes filled with water, η is fixed at the value obtained in the absence of tubes and water, and the value of *t* is evaluated from the fit. In particular, we note that the obtained value, that is t=0.56±0.02, is compatible with the expected values of transmittance efficiency due to each optical element (0.93) and water absorption (α=0.04 m${}^{-1}$ and x=1.7 m) [15]. Thanks to the good quality of data, as a second kind of investigation, we consider the feasibility of the communication protocol mentioned in the Introduction and based on the use of TWB on which a binary thermal noise signal is superimposed [20]. Indeed, pseudothermal states of light can be obtained by passing the laser beam, such as the second harmonic of the Nd:YLF laser mentioned in the previous section, through a rotating ground-glass disk and selecting a single speckle from the obtained speckle pattern by means of a pin-hole. The general scheme of the optical communication link is shown in Figure 6.

To mimic the effect of superimposing a pseudothermal signal state on one arm of TWB, we process the data offline.

The procedure can be outlined as follows:

- Consider the data for the two arms of the TWB shown as gray dots in Figure 5 ($m_{1}$, free space arm and $m_{2}$, through-water arm);
- Consider the data of the pseudothermal noise signal $m_{N}$ for two different mean values, called high and low mean values hereafter;
- Sum the measured single-shot values of the pseudothermal state with “low” mean value to the values of the TWB arm that passed through water;
- Evaluate $R=\sigma^{2}\left(m_{2}+m_{N}-m_{1}\right)/\langle m_{1}+m_{2}+m_{N}\rangle$ using the resulting values;
- Repeat the procedure for the “high” mean value pseudothermal noise.

**Figure 7:**
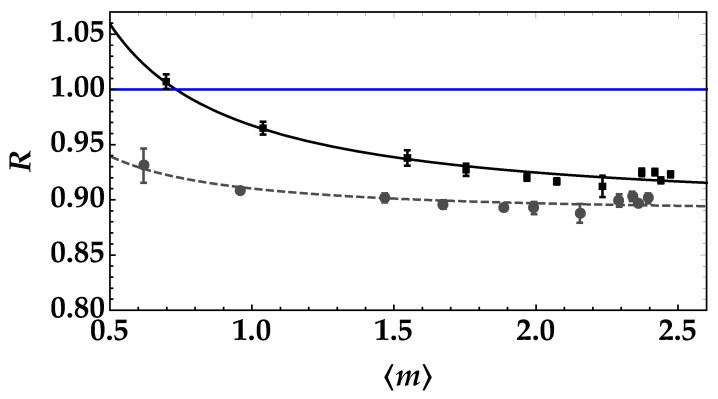
Noise reduction factor as a function of the mean number of detected photons in each arm for the pseudothermal noise signal with the highest mean value (black squares + error bars) and for the pseudothermal noise signal with the lowest mean value (gray dots + error bars). The theoretical fitting function according to Equation (5) is superimposed on each data set with the same color choice. The values of the fitting parameters for black data are: t=0.55±0.03 and $\langle m_{N}\rangle =0.32\pm 0.01$, while those corresponding to gray data are: t=0.55±0.02 and $\langle m_{N}\rangle =0.17\pm 0.02$. The blue straight line corresponds to the condition R=1.

It is worth noting that the two mean values (high and low) of the pseudothermal noise signal should be smaller (roughly by a factor of 10) than the mean value of TWB in order to preserve nonclassicality. The two values must be chosen in a such a way that they can be discriminated by the evaluation of the noise reduction factor. In Ref. [20], we have demonstrated that if the lowest mean value of noise is ∼3–4 times smaller than the highest one, the discrimination of the two states is also possible in the presence of an eavesdropper’s attack. The resulting values of the noise reduction factors are shown in Figure 7, where the black squares + error bars refer to the superposition of the high-mean value pseudothermal signal, and gray dots + error bars to the superposition of the low-mean value signal. If we fit each dataset according to Equation (5), we can obtain the values of *t* and $\langle m_{N}\rangle$ as fitting parameters. In particular, we obtain: t=0.55±0.03 and $\langle m_{N}\rangle =0.32\pm 0.01$ for the highest pseudothermal signal (black squares), and t=0.55±0.02 and $\langle m_{N}\rangle =0.17\pm 0.02$ for the lowest pseudothermal signal (gray dots). We emphasize that the values of *t* are in perfect agreement to each other and also with the value of *t* obtained from the fit of the data in Figure 5, thus proving that the operated processing of data does not change the characteristics of the transmission channel. As a second observation, we note that the obtained mean values of the pseudothermal signals are in agreement with their direct determination from the strings of data. All these facts prove the validity and completeness of our theoretical model.

The next step in view of implementing the communication strategy consists of choosing the mean value of the TWB on which the two pseudothermal signals can be alternately superimposed. Moreover, it is necessary to investigate the minimum length of data sets that allows discriminating the two superimposed pseuthermal signals. Note that the evaluation of the mean value is sufficient for the discrimination only in the absence of noise and/or losses introduced by an eavesdropper. On the contrary, as shown in a previous paper of ours [20], in the presence of added noise and/or losses, measuring the mean value is no longer a reliable discrimination strategy, while the calculation of the noise reduction factor still remains a valid strategy because all the parameters (〈m〉, $\langle m_{N}\rangle$, and *t*) explicitly appear in its expression (see Equation (4)). However, in that work, we did not consider a realistic situation, while in this case, we can test the strategy in the presence of a real imbalance between the two arms due to propagation in water. Thus, it is of crucial importance to investigate what happens to the discrimination strategy if data samples of different lengths and corresponding to one of the two mean values of pseudothermal signals are used.

**Figure 8:**
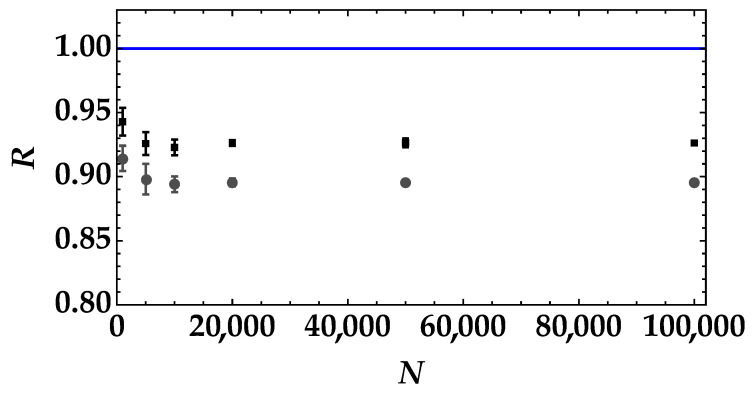
Noise reduction factor as a function of the number of data samples for the pseudothermal noise signal with the highest mean value (black squares + error bars) and for the pseudothermal noise signal with the lowest mean value (gray dots + error bars). The blue straight line corresponds to the condition R=1.

In Figure 8, we show the noise reduction factor as a function of the number of data for the pseudothermal signals with the highest mean value (black data + error bars) and the lowest mean value (gray data + error bars). In both cases, the mean number of detected photons of the TWB is equal to 2.037±0.006. We can notice that a data sample equal to 1000 shots is enough to discriminate the two states even if the error bars decrease at increasing sample size.

**Figure 9:**
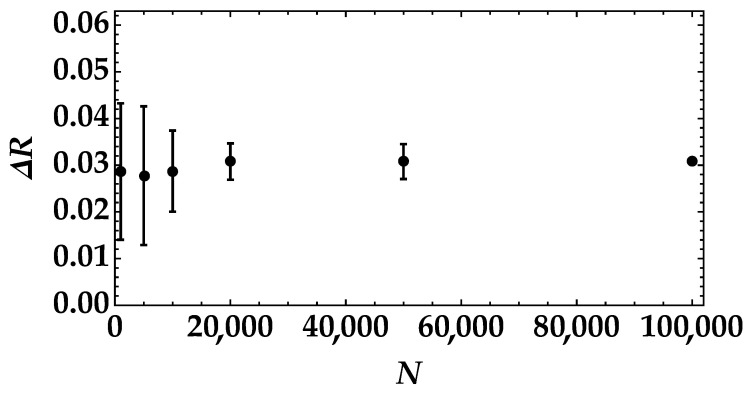
Difference between the noise reduction factor in the case of the highest mean value of pseudothermal signal and the one in the case of the lowest mean value as a function of the number of data samples.

This result can be further appreciated by plotting the difference between the noise reduction factor in the case of the highest mean value of pseudothermal noise and the one in the case of the lowest mean value (see Figure 9). We can clearly see that the value of ΔR has larger error bars for N=1000 than for larger amounts of data samples, such as for N>20,000.

## 4. Discussion

In this work, we investigated the possibility of using mesoscopic TWB states to perform underwater quantum communication in the case in which one arm of the TWB is transmitted in free space, while the other one propagates partly in free space and partly in water. In particular, we considered three different Jerlov types of water. Actually, the difference between the three types of water considered is negligible, since the values of the absorption coefficient per unit length are quite similar. The obtained results could be improved considering a wavelength in the blue region instead of in the green one, since in that case, the absorption coefficient per unit length is smaller. Moreover, we investigated the robustness of nonclassicality to the presence of a pseudothermal noise superimposed on the arm of TWB transmitted in water. The threshold R=1 depends both on the mean value of TWB and on the amount of loss due to water absorption. Moreover, low values of the noise reduction factor can be attained by decreasing the mean value of the pseudothermal noise as well as shortening the propagation distance in water. From the experimental point of view, we considered true propagation in Jerlov type I water and proved that the losses due to water absorption and to the presence of some optical elements can be perfectly explained through the developed theoretical model. Moreover, we proved that the same holds in the case of a binary pseudothermal state superimposed on the arm of TWB propagating in water. As expected, the larger the noise, the higher the value of the noise reduction factor, and thus, the lower the nonclassicality level. In view of implementing the communication protocol, in which the information is encoded in the pseudothermal noise, we checked the minimum length that allowed us to properly discriminate which signal state was sent. The results are in agreement with the theoretical findings shown in a previous work of ours [20] and suggest that a more realistic implementation of the protocol can be performed. This can open new perspectives in the communication context, where the typical degrees of freedom used to encode information are represented by polarization, orbital angular momentum, transverse momentum, and time-bins [32]. On the contrary, here, the encoding involves photon numbers, since we exploit the photon number resolution of our detectors and the noise reduction factor, which is a nonclassicality criterion based on photon numbers. Further improvements toward a more realistic implementation of the protocol could be the use of more portable PNR detectors, such as silicon photomultipliers [33,34], as already stated in Ref. [15]. Moreover, shifting the light propagating in water to the blue region can make the nonclassicality preservation easier, since the absorption coefficient per unit length is smaller. This implies either the change of the wavelength of the pump beam itself or the selection of a non-degenerate TWB state. To this aim, the choice of silicon photomultipliers as the detectors could be crucial, since there are models optimized in specific spectral regions [35].

## 5. Conclusions

The possibility of performing an underwater communication protocol, in which a binary pseudothermal signal is superimposed on one arm of TWB, has been investigated both theoretically and experimentally. The obtained results are very promising in view of a more realistic implementation of the protocol. More improvements can be also obtained by using a different class of PNR detectors, such as silicon photomultipliers, instead of the employed HPDs. Indeed, the sensitivity peak of the former detectors can be tuned from the near ultraviolet up to the near infrared, thus allowing the detection of non-degenerate TWB states. Moreover, they are endowed with a good dynamic range so that they can be employed to detect optical states with mean values as large as tens of photons. This feature makes it possible to operate in the truly mesoscopic intensity regime.

## Data Availability

Data underlying the results presented in this paper are not publicly available at this time but may be obtained from the author upon reasonable request.

## Abbreviations

The following abbreviations are used in this manuscript:

TWB	twin-beam
PNR	photon number resolving
HPD	hybrid photodetector
